# Efficacy and safety of extended depth of focus intraocular lenses in cataract surgery: a systematic review and meta-analysis

**DOI:** 10.1186/s12886-019-1204-0

**Published:** 2019-09-02

**Authors:** Jing Liu, Yi Dong, Yan Wang

**Affiliations:** 1 Clinical College of Ophthalmology, Tianjin Medical University, No 4. Gansu Rd, Heping District, Tianjin, 300020 China; 2 Tianjin Key Laboratory of Ophthalmology and Visual Science, Tianjin Eye Hospital, Tianjin Eye Institute, No 4. Gansu Rd, Heping District, Tianjin, 300020 China

**Keywords:** Extended depth of focus, Trifocal, Monofocal, Intraocular lens, Cataract, Visual function, Meta-analysis

## Abstract

**Background:**

This study aims to evaluate the efficacy and safety of extended depth of focus (EDOF) intraocular lenes (IOLs) in cataract surgery.

**Methods:**

All comparative clinical trials that involved bilaterally implanting EDOF IOLs in patients with cataract were retrieved from the literature database. We used random effects models to pool weighted mean differences (WMD) and risk ratio (RR) for continuous and dichotomous variables, respectively.

**Results:**

Nine studies with a total of 1336 eyes were identified. The subgroup analysis was conducted according to the type of IOLs used in the control group. Compared with monofocal IOLs, EDOF IOLs produced better uncorrected intermediate visual acuity (WMD: -0.17, 95% CI: − 0.26 to − 0.08, *P* = 0.0001) and uncorrected near visual acuity (WMD: -0.17, 95% CI: − 0.21 to − 0.12, *P* < 0.00001). EDOF IOLs resulted in reduced contrast sensitivity, more frequent halos, however, higher spectacle independence (RR: 2.81, 95% CI: 1.06 to 7.46, *P* = 0.04) than monofocal IOLs. Compared with trifocal IOLs, EDOF IOLs produced worse near visual acuity (MD: 0.10, 95% CI: 0.07 to 0.13, *P* < 0.0001). EDOF IOLs performed better than trifocal IOls in contrast sensitivity, and there were no significant difference in halos and spectacle independence. Serious postoperative complications were rare, with no adverse events were reported in most studies.

**Conclusions:**

Increasing the risk of contrast reduction and more frequent halos, EDOF IOLs provided better intermediate and near VAs than monofocal IOLs. At the expense of near vision, patients receiving EDOF IOLs have better contrast sensitivity than those receiving trifocal IOLs. Halo incidence and spectacle independence of EDOF IOLs were similar to those of trifocal IOLs.

## Background

Monofocal intraocular lenses (IOLs) are the most commonly implanted IOLs in cataract surgery [1]. With a single focal point, monofocal IOLs are effective in restoring satisfactory distance vision; however, most patients require spectacle correction for intermediate and near vision, even after surgery [1, 2]. Thus, multifocal IOLs were designed to meet the increasing demand from patients for spectacle independence [3]. For providing far, intermediate and near vision simultaneously, multifocal IOLs possess two or more independent focal points, which result in contrast reduction and increased photic phenomena, thus reducing visual quality [4].

More recently, a new-concept IOL was introduced based on extended depth of focus (EDOF) technology [5]. The basic principle behind EDOF IOLs is to create a single elongated focal point to enhance the depth of focus or range of vision [6]. A proprietary diffractive echelette design is used in EDOF IOLs and forms a step structure. The height, spacing, and profile of the echelettes are optimized to achieve constructive interference of light from different lens zones, thus producing a novel light diffraction pattern. In addition, proprietary achromatic technology and negative spherical aberration correction improve the image quality [7]. With technological advancement, EDOF IOLs showed good visual outcomes with less contrast reduction and fewer photic phenomena commonly associated with multifocal IOLs [4, 8]. However, according to some studies, EDOF lenses worked less efficiently for near vision than did trifocal IOLs [9, 10]. Currently, several types of EDOF IOLs are commercially available, including the Tecnis Symfony (Johnson and Johnson Vision), Mini WELL (Sifi Medtech), IC-8 (AcuFocus Inc) and Wichterle Intraocular Lens-Continuous Focus (Medicem). Until 2018, the Tecnis Symfony was the only United States Food and Drug Administration (FDA)-approved EDOF lens [6].

Although many studies have been conducted to characterize the efficacy and safety of EDOF IOLs, the unique features, such as visual acuity, vision quality and complications of EDOF IOLs is less clear-cut. Thus, we performed a systematic review and meta-analysis of randomized and nonrandomized controlled studies (NRCSs) to compare the clinical performance of EDOF IOLs with that of monofocal and trifocal IOLs. Finally, our study used only Tecnis Symfony IOL as the representative of EDOF IOLs due the lack of studies on other EDOF lenses.

## Methods

### Search strategy

The PubMed, EMBASE, Web of Science, ClinicalTrials.gov and Cochrane Library databases (most recently updated in 2019 January) were searched using the keywords “extended depth of focus intraocular lens”, “extended range of vision intraocular lens” and “cataract surgery”. No language limitations were applied in the search strategy. In addition, the references of identified articles and reviews were checked and matching publications were included. Two reviewers (J. L. and Y. D.) independently conducted searches and scanned the abstracts, followed by full-text articles to determine whether the articles met the eligibility criteria. A third reviewer (Y. W.) was consulted when disagreement existed between J. L. and Y. D.

### Eligibility criteria

We included all clinical controlled studies (randomized or nonrandomized, from 2000 to 2019 January) comparing clinical outcomes of EDOF IOLs with those of control IOLs in patients undergoing cataract surgery. However, studies involving patients with previous refractive surgery, irregular or > 1.0 diopter (D) corneal astigmatism and coexisting pathology, such as amblyopia, keratoconus, corneal endothelial dystrophy, chronic or recurrent uveitis, acute ocular disease or external/internal infection, diabetes mellitus with retinal changes, glaucoma and choroidal hemorrhage were excluded. We also excluded studies with double implantation in the same eye, no bilateral implantation, double reporting, in vitro experiments and no aggregated results.

### Qualitative assessment and data extraction

The Jadad [11] and Newcastle-Ottawa Scale (NOS) [12] were used to assess the quality of randomized controlled trials (RCTs) and NRCSs, respectively. The maximum NOS score is nine points, and a score over six points indicates good quality. Two reviewers (J. L. and Y. D.) independently extracted the characteristic data of included studies using a standard form; we tried to contact the author for sufficient information and original data when necessary. Discrepancies between two reviewers were resolved by a third reviewer (Y. W.).

### Outcome measures

Primary outcomes included binocular uncorrected distance visual acuity (UDVA), uncorrected intermediate visual acuity (UIVA), uncorrected near visual acuity (UNVA), defocus curves and contrast sensitivity. Visual acuity was evaluated using the high-contrast Early Treatment Diabetic Retinopathy Study chart in logMAR units under photopic conditions. Binocular defocus curves were done with best distance correction. Different levels of defocus were introduced in 0.50 D steps from + 1.00 to − 4.00 D. Contrast sensitivity under photopic and scotopic conditions for 1.5, 3.0, 6.0, 12.0, and 18.0 cycles per degree. The contrast sensitivity data was difficult to pool because of the considerable variety of the measurement methods. Thus, contrast sensitivity was instead reported descriptively. Halos, spectacle independence and postoperative complications were defined as the secondary outcomes. Spectacle independence was obtained from self-reported questionnaires and defined as the proportion of subjects who reported wearing glasses or contacts “none of the time” or “a little of the time” for overall vision.

### Statistical analysis

We used RevMan software (version 5.3, Cochrane Collaboration) to analyze the data. The weighted mean difference (WMD) and risk ratio (RR) with 95% confidence interval (CI) were calculated for continuous and dichotomous variables, respectively. A *P*-value < 0.05 was defined as statistically significant. Forest plots were used to present the results. In forest plots, only subtotals were analyzed because of the evident difference in design principles between monofocal and trifocal IOLs in control groups. Green boxes indicate the mean value, and the size of boxes indicates the weighting given to that estimate. The 95% CI for the estimate is shown as a horizontal line. The diamond represents the mean effect size. The center of the diamond represents the pooled point estimate, and the horizontal tips show the CI. We chose the random effects model for all data analyses because studies differed in trial design, patient ages, implanted IOLs, and the longest follow-up time. For multiarm studies, we combined groups to create a single pairwise comparison as recommended by the Cochrane Handbook for Systematic Reviews of Interventions [13]. To verify the stability of the results, we performed sensitivity analysis by individually omitting the included studies. Publication bias was measured visually using funnel plots.

### Heterogeneity management

Statistical heterogeneity was tested by I^2^ tests [14]. Findings were considered statistically significant if I^2^ > 50%. Under the assumption that the type of IOLs would explain a portion of heterogeneity, subgroups were defined as monofocal IOLs and trifocal IOLs in control groups.

## Results

### Result of the search

**Fig. 1:**
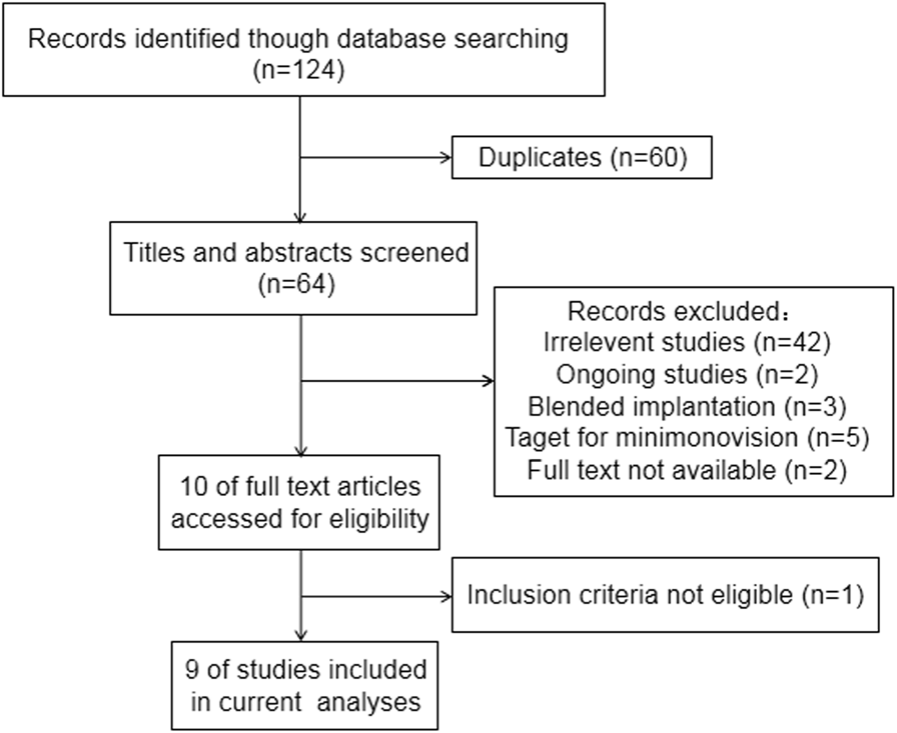
Flow chart showing the study selection process

The electronic searches identified a total of 124 records. Figure 1 shows a flow diagram of the included and excluded studies. Two conference abstracts were excluded because the full text was unavailable [15, 16]. We tried to contact the author but did not receive a reply. Of 10 studies potentially relevant for this meta-analysis, one study enrolling patients with preexisting corneal astigmatism of 1.00 D or worse was excluded [17]. Ultimately, 9 studies were included in our quantitative analysis [9, 10, 18–24].

### Study characteristics and quality

Table 1 summarizes the characteristics and quality of the 9 studies that met all inclusion criteria [9, 10, 18–24]. Of the 9 selected studies, 3 were RCTs and 6 were NRCSs with a total of 1336 eyes. The studies were performed in various countries, and all studies were published between 2016 and 2018. The RCT sponsored by Abbott Medical Optics (AMO) company, leaded to the U.S. FDA approval of Tecnis Symfony IOL in 2016 [24]. Tecnis Symfony ZXR00 was used in the EDOF IOL group, while monofocal IOLs (Tecnis ZCB00 and AcrySof SN60WF) and trifocal IOLs (PanOptix, FineVison and Lisa tri 839MP) were used in the control groups. The follow-up period ranged from 3 to 29 months. The Jadad method was used to assess the methodological quality of RCTs in 3 respects: randomization, blindness and dropouts. Two of three RCTs were scored higher than 3 points. All six NRCSs were of relatively low risk of bias, scoring higher than 6 points on the NOS.

**Table 1:** Characteristics and quality of included studies

Study^a^, year	Location	Design	IOL	No. of patients/eyes	Longest follow up (month)	Jadad	Newcastle-Ottawa Scale
Cochener, 2018 [9]	France	RCT	Symfony PanOptix FineVison	20/40 20/40 20/40	6	Randomization 1 Blindness 1 Dropouts 1	–
Mencucci, 2018 [10]	Italy	NRCS	Symfony PanOptix Lisa tri 839MP	20/40 20/40 20/40	3	–	Selection 3 Comparability 2 Outcome 2
AMO, 2017 [24]	United States	RCT	Symfony ZCB00	148/296 151/302	6	Randomization 1 Blindness 2 Dropouts 1	–
Escandón-García, 2018 [18]	Portugal	NRCS	Symfony PanOptix FineVison	15/30 7/14 23/46	3	–	Selection 3 Comparability 1 Outcome 2
Monaco, 2017 [19]	Italy	RCT	Symfony PanOptix SN60WF	20/40 20/40 20/40	4	Randomization 2 Blindness 1 Dropouts 1	–
Pedrotti, 2016 [20]	Italy	NRCS	Symfony ZCB00	25/50 15/30	3	–	Selection 3 Comparability 2 Outcome 3
Pilger, 2018 [21]	Germany	NRCS	Symfony ZCB00	15/30 15/30	3	–	Selection 3 Comparability 2 Outcome 3
Ruiz-Mesa, 2017 [22]	Spain	NRCS	Symfony FineVison	20/40 20/40	12	–	Selection 3 Comparability 2 Outcome 2
Ruiz-Mesa, 2018 [23]	Spain	NRCS	Symfony PanOptix	14/28 20/40	29	–	Selection 2 Comparability 2 Outcome 2

*AMO* Abbott Medical Optics, *IOL* intraocular lens, *RCT* randomized controlled trial, *NRCS* non-randomized controlled study

^a^First author or sponsor

### Primary outcomes

#### Binocular uncorrected visual acuity

**Fig. 2:**
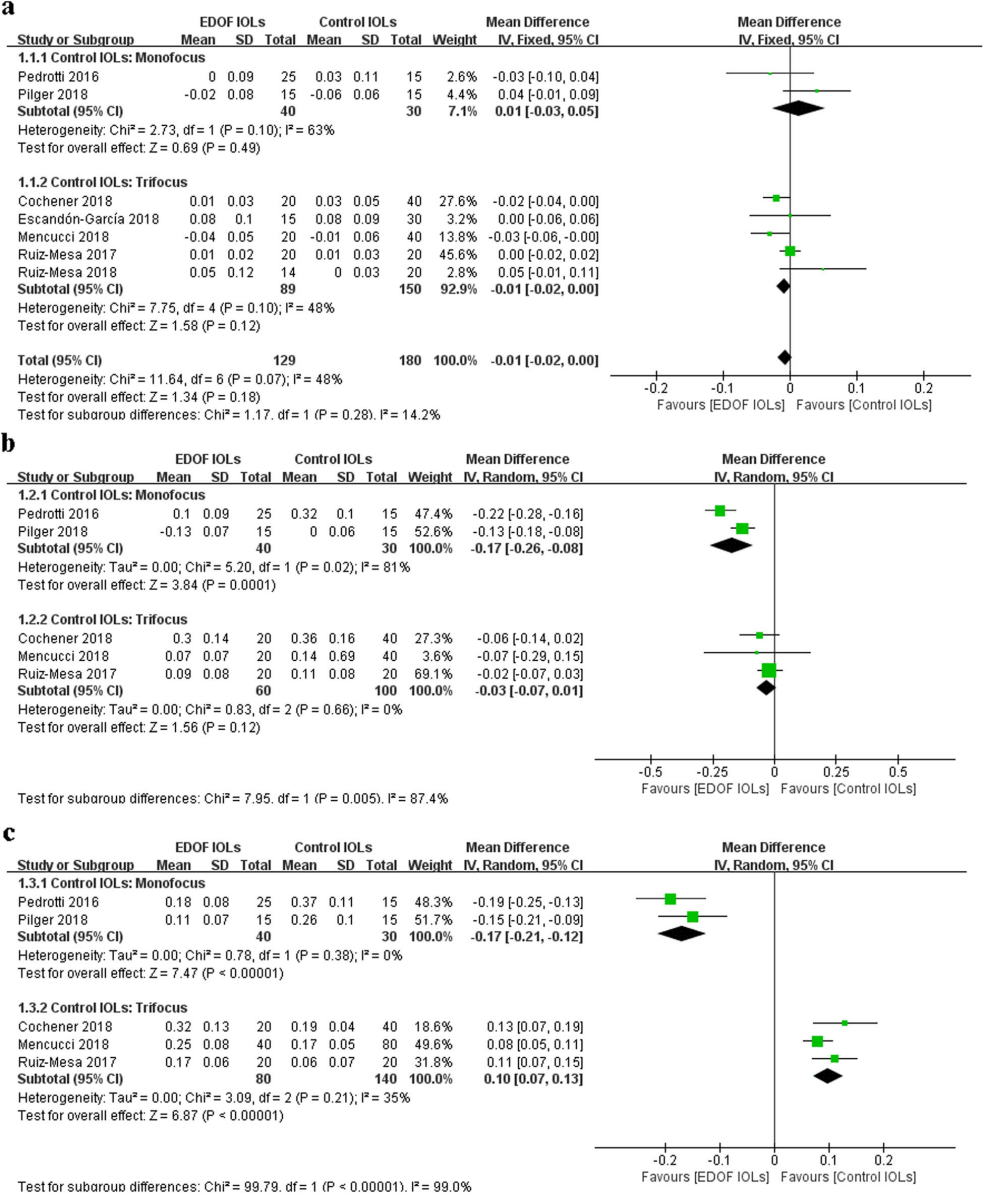
Forest plot of binocular uncorrected visual acuity. **a** UDVA. **b** UIVA. **c** UNVA

Seven [9, 10, 18, 20–23], five [9, 10, 20–22] and five [9, 10, 20–22] studies reported binocular UDVA, UIVA and UNVA, respectively (Fig. 2). One study did not report the standard deviation (SD) or other data to calculate the SD and thus was excluded from the analysis [24]. We tried to contact the author but did not receive a reply. The subgroup analysis was conducted according to the type of IOLs used in the control group. Compared with monofocal IOLs, EDOF IOLs provided comparable UDVA (WMD: 0.01, 95% CI: − 0.06 to 0.08, *P* = 0.81), better UIVA (WMD: -0.17, 95% CI: − 0.26 to − 0.08, *P* = 0.0001) and better UNVA (WMD: -0.17, 95% CI: − 0.21 to − 0.12, *P* < 0.00001). Compared with trifocal IOLs, EDOF IOLs showed no significant differences in UDVA (WMD: -0.01, 95% CI: − 0.03 to 0.01, *P* = 0.34) or UIVA (WMD: -0.03, 95% CI: − 0.07 to 0.01, *P* = 0.12) and performed worse in UNVA (WMD: 0.10, 95% CI: 0.07 to 0.13, *P* < 0.0001). In sensitivity analysis, no single study significantly changed the pooled estimate, indicating that the results were stable.

#### Defocus curves

**Fig. 3:**
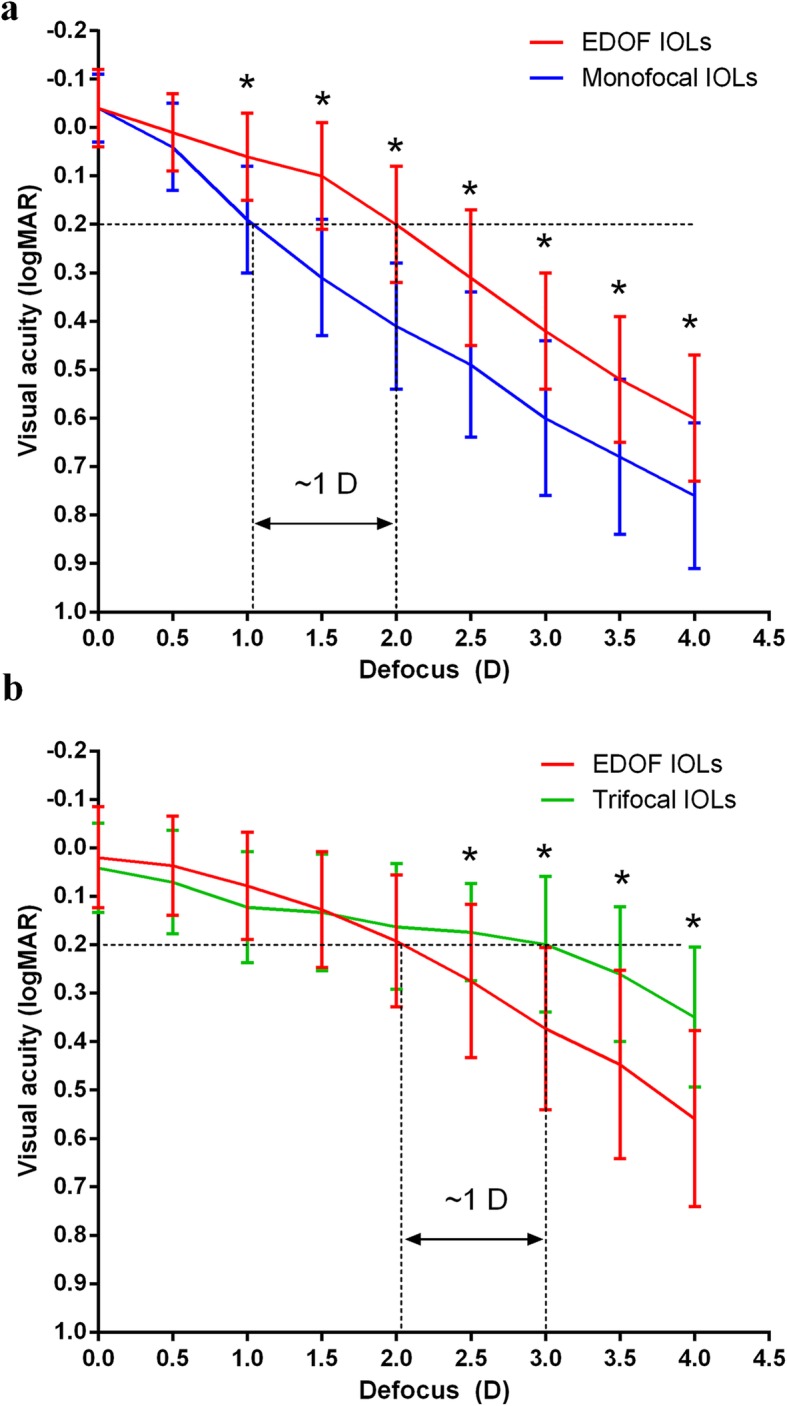
Defocus curves. **a** EDOF and monofocal IOLs. **b** EDOF and trifocal IOLs

Six studies [18–20, 22–24] reported binocular distance-corrected defocus curves. The binocular defocus curves based on 3 trails of 215 subjects for EDOF and monofocal IOLs and 4 trails of 159 subjects for EDOF and trifocal IOLs are shown in Fig. 3. Monofocal, EDOF and trifocal IOLs sustained 0.2 logMAR or better mean VA through 1.0 D, 2.0 D and 3.0 D, respectively. VA was significantly better with EDOF IOLs than with monofocal IOLs in the defocus levels from − 1.0 to − 4.0 D. VA was significantly better in trifocal IOL group than in EDOF IOL group from − 2.5 to − 4.0 D (Table 2). The sensitivity analysis showed that no single study significantly changed the pooled estimate, indicating the results of defocus curves were stable.

**Table 2:** Results of Meta-analysis for Defocus Curve

Defocus levels	MD [95% CI]	*P* value	Heterogeneity	
			I^2^ (%)	*P*_*heterogeneity*_
EDOF vs. Monofocal IOLs				
-0.01	−0.00 (− 0.10, 0.08)	0.81	89	0.0001
−0.50	−0.04 (− 0.09, 0.00)	0.07	25	0.26
−1.00	−0.16 (− 0.21, − 0.12)	< 0.00001	0	0.65
− 1.50	− 0.22 (− 0.31, − 0.13)	< 0.00001	63	0.07
−2.00	− 0.24 (− 0.29, − 0.19)	< 0.00001	8	0.34
− 2.50	− 0.22 (− 0.27, − 0.16)	< 0.00001	0	0.45
− 3.00	− 0.25 (− 0.31, − 0.18)	< 0.00001	37	0.20
−3.50	− 0.21 (− 0.26, − 0.16)	< 0.00001	0	0.94
−4.00	−0.21 (− 0.26, − 0.16)	< 0.00001	0	0.73
EDOF vs. Trifocal IOLs				
0.00	−0.02 (− 0.07, 0.03)	0.40	59	0.06
−0.50	−0.03 (− 0.08, 0.01)	0.17	52	0.10
−1.00	−0.04 (− 0.10, 0.01)	0.11	55	0.08
−1.50	−0.01 (− 0.08, 0.07)	0.88	76	0.006
−2.00	0.03 (−0.01, 0.07)	0.19	0	0.96
−2.50	0.10 (0.06, 0.15)	< 0.00001	0	0.79
−3.00	0.17 (0.09, 0.26)	< 0.0001	65	0.04
−3.50	0.19 (0.07, 0.30)	0.002	68	0.04
−4.00	0.21 (0.07, 0.35)	0.003	79	0.008

*IOL* intraocular lens, *MD* mean difference, *CI* confidence interval, *I*^*2*^ extent of inconsistency

#### Contrast sensitivity

Seven studies [10, 18, 20–24] reported contrast sensitivity and the results are summarized in Table 3. The U.S. FDA clinical trial reported that the median contrast scores for the EDOF IOL group were reduced compared to the monofocal control group under both conditions and each spatial frequency [24]. Pilger et al. reported that EDOF IOLs performed worse than did monofocal IOLs under scotopic conditions [21]. Pedrotti et al. reported no significant difference in contrast sensitivity between EDOF and monofocal IOLs under both photopic and scotopic conditions [20]. Mencucci et al. reported that EDOF performed significantly better than trifocal IOLs under both photopic and scotopic conditions [10]. Escandón-García et al. reported that EDOF IOLs performed better than trifocal IOLs at a frequency of 1.5 cycles per degree under scotopic conditions [18]. Two studies reported no difference in contrast sensitivity between EDOF and trifocal IOLs [22, 23].

**Table 3:** Summary of Contrast Sensitivity and Halos

Study^a^, year	EDOF IOLs	Control IOLs	CS: Under photopic conditions	CS: Under scotopic conditions	Halos
Pedrotti, 2016 [20]	Tecnis Symfony	Tecnis ZCB00	NSD	NSD	NSD
AMO, 2017 [24]	Tecnis Symfony	Tecnis ZCB00	Better in monofocal IOLs group	Better in monofocal IOLs group	More halos in EDOF IOLs group
Pilger, 2018 [21]	Tecnis Symfony	Tecnis ZCB00	NR	Better in monofocal IOLs group	NSD
Cochener, 2018 [9]	Tecnis Symfony	PanOptix/ FineVison	NR	NR	NSD
Escandón-García, 2018 [18]	Tecnis Symfony	PanOptix/ FineVison	NSD	For 1.5 cpd, better in EDOF IOLs group	NR
Mencucci, 2018 [10]	Tecnis Symfony	PanOptix/AT LISA tri 839MP	Better in EDOF IOLs group	Better in EDOF IOLs group	NSD
Monaco, 2017 [19]	Tecnis Symfony	PanOptix/SN60WF	NR	NR	EDOF verses trifocus: NSD; Both were worse than monofocal IOL
Ruiz-Mesa, 2017 [22]	Tecnis Symfony	FineVison	NSD	NSD	NSD
Ruiz-Mesa, 2018 [23]	Tecnis Symfony	PanOptix	NSD	NSD	NSD

*AMO* Abbott Medical Optics, *EDOF* extended depth of focus, *CS* contrast sensitivity, *IOLs* intraocular lenses, *cpd* cycles per degree, *NSD* no significant difference, *NR* not report

^a^First author or sponsor

### Secondary outcomes

#### Halos

Eight studies [9, 10, 19–24] used questionnaires and Halo software to record halos. Because these studies used different questionnaires and measurements, conducting quantitative analyses of halos was inappropriate. Instead, the results are descriptively summarized in Table 3. Two studies reported no significant difference in halos between EDOF and monofocal IOLs [20, 21]. The U.S. FDA clinical trial reported that EDOF IOLs resulted in more frequent halos than monofocal IOLs [24]. Monaco et al. reported that both EDOF and trifocal IOLs resulted in more frequent halos than did monofocal IOLs [19]. Five studies reported no difference in halos between EDOF and trifocal IOLs [9, 10, 19, 22, 23].

#### Spectacle Independence

**Fig. 4:**
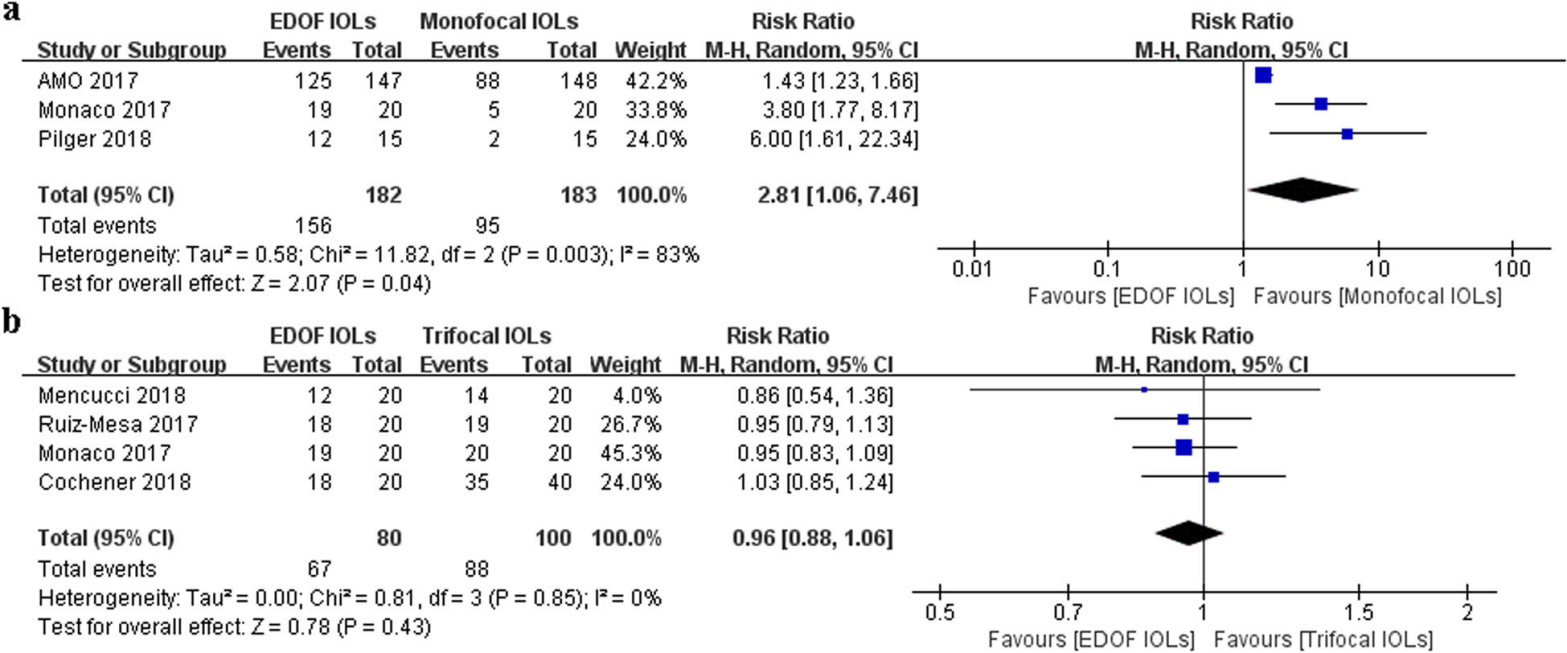
Forest plot of spectacle independence. **a** EDOF and monofocal IOLs. **b** EDOF and trifocal IOLs

Six studies [9, 10, 19, 21, 22, 24] reported spectacle independence. There was a significant difference in the overall effect that favored higher spectacle independence with EDOF IOLs than with monofocal IOLs (RR: 2.81, 95% CI: 1.06 to 7.46, *P* = 0.04) (Fig. 4a). The studies were characterized by high heterogeneity (I^2^ = 83%, *P* = 0.003). There was no significant difference between EDOF and trifocal IOLs in the overall effect (RR: 0.96, 95% CI: 0.85 to 1.07, *P* = 0.45) (Fig. 4b). No significant heterogeneity was found (I^2^ = 0%, *P* = 0.61).

#### Postoperative complications

Two studies [22, 24] reported postoperative complications of EDOF IOLs. The complication reported in the U.S. FDA clinical trial included a rate of 1.35% for cystoid macular edema, 0.68% for pupillary capture, 0.68% for endophthalmitis and 0.68% for hypopyon 6 months postoperatively [24]. One study reported 0 and 5% of patients had posterior capsule opacification 12 months postoperatively in the EDOF IOL group and trifocal IOL group respectively [22].

### Publication Bias

**Fig. 5:**
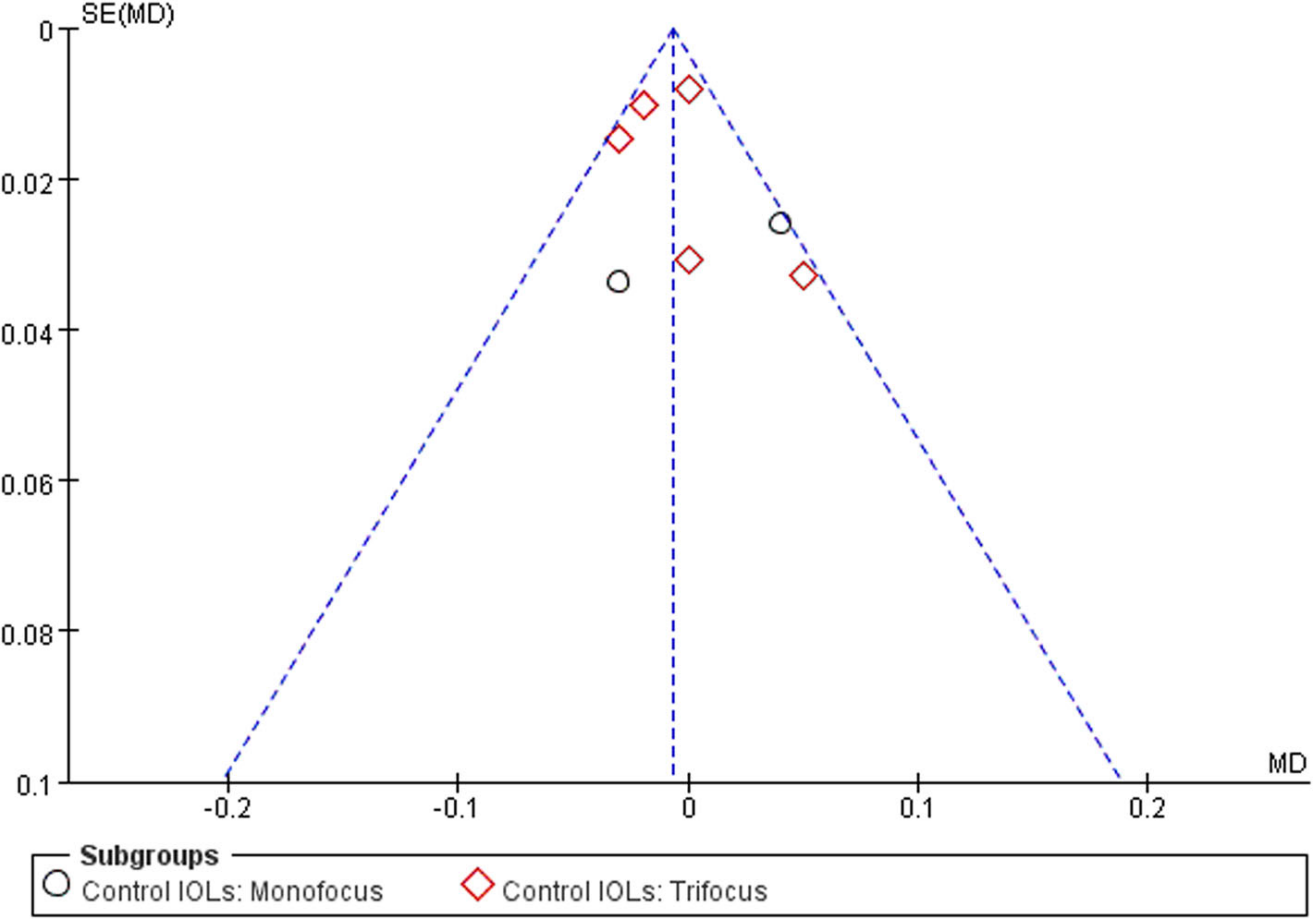
Funnel plot for publication bias test

The publication bias of the studies was determined by a funnel plot. The symmetrical funnel plot showed no significant publication bias in the publications (Fig. 5).

## Discussion

The present meta-analysis compared the clinical performance of EDOF IOLs with those of monofocal and trifocal IOLs. According to the results, compared with monofocal IOLs, EDOF IOLs have benefits for intermediate and near vision, but also increase the risk of contrast reduction and more frequent halos. Although EDOF IOLs worked less efficiently for near vision than did trifocal IOLs, they maintained better contrast sensitivity and no differences were found in halo incidence and spectacle independence.

All studies included in this meta-analysis involved bilateral implantation. Implantation of the same IOLs in both eyes avoids overestimating or underestimating the efficacy of the IOL caused by interference from the follow eye. Therefore, bilateral implantation is a more effective way to measure the effect of IOLs on quality of life [25].

Creating a single elongated focal point to enhance the range of vision, EDOF IOLs expectedly provided better uncorrected intermediate and near VA than that of monofocal IOLs [6]. However, EDOF IOLs performed worse on near vision than did trifocal IOLs that splits light into distant, intermediate and near focal points. So the near vision of EDOF IOLs is somewhere between that of monofocal and trifocal IOLs. EDOF IOLs and trifocal IOLs performed similarly on distance and intermediate visions. To reflect vision-related quality of life more directly, uncorrected VAs, instead of corrected VAs were main vision outcomes in our meta-analysis [26].

Binocular defocus curves also showed comparable distance and intermediate visions with EDOF and trifocal IOLs and better near vision with trifocal IOLs. Although EDOF IOLs improved the range of defocus with VAs of 0.2 logMAR or better by approximately 1 D than monofocal IOLs, trifocal IOLs had the longest range of defocus from 0 to − 3.0 D (VA above 0.2 logMAR). Therefore, EDOF IOLs had superior visual outcomes between 1 m and 25 cm than monofocal IOLs and inferior visual outcomes between 40 cm and 25 cm than trifocal IOLs. Based on the results of VAs and defocus curves, the EDOF IOL provides excellent distance and intermediate vision but mediocre near vision.

All monofocal IOLs involved in the current study were aspherical. Aspherical monofocal IOLs have been reported to provide higher contrast sensitivity than spherical IOLs and multifocal IOLs [27]. Although the Symfony EDOF IOL employed achromatic and aspheric technologies to maintain visual quality [6], it caused a reduction in contrast sensitivity compared to aspherical monofocal IOLs. With EDOF IOLs, there is a tradeoff between the clarity of near vision and contrast sensitivity. However, the present study found that the contrast sensitivity of EDOF IOLs was higher than that of trifocal IOLs, especially under scotopic conditions [10, 18]. In trifocal IOLs, the distribution of light to more than one focus results in contrast reduction postoperatively, one of the major limitations of multifocal IOLs [4].

Depending on the difference in individual habits and lifestyle in real contexts, spectacle independence is a subjective parameter. Although EDOF IOLs worked less efficiently for near vision than did trifocal IOLs, there was no difference between EDOF and trifocal IOLs in self-reported spectacle independence. In addition, there was no difference in halo incidence between the two groups. This may be explained by the fact that most patients are capable of adapting and tend to become more tolerant of photic phenomena several months postoperatively [28].

Serious postoperative complications were rare and most of studies did not routinely include complications in their outcome measures. One study reported that trifocal IOLs induced more posterior capsule opacification than EDOF IOLs 12 months postoperatively [22]. More studies are needed to prove the safety of EDOF IOLs.

To our knowledge, this meta-analysis is the first to compare the clinical performance of EDOF IOLs in cataract surgery with that of monofocal and trifocal IOLs, respectively. However, this meta-analysis has several limitations. First, between-study heterogeneity was substantial. The included studies varied in length of follow-up, types of IOLs in the control group, study location and measurement methods. We chose the random model for all data analyses and tried to explain the heterogeneity by subgroup analyses and sensitivity analyses. The results were stable in sensitivity analyses by individually omitting the included studies. Second, only 3 of the included studies were RCTs, and the remaining studies were NRCSs that had a potential selection bias. Third, publication bias was suspected due to the exclusion of unpublished studies and conference abstracts. Last, limited number of studies reported postoperative complications. More clinical trails that record postoperative adverse effects are needed to assess the safety of EDOF IOLs.

## Conclusions

This systematic review revealed the unique features of EDOF IOLs when compared with other types of IOLs. Compared with monofocal IOLs, EDOF IOLs have benefits for intermediate and near vision but also increase the risk of contrast reduction and more frequent halos. Compared to trifocal IOLs, EDOF IOLs worked less efficiently for near vision; however, this limitation may be an acceptable compromise to patients, given the accompanying retained contrast sensitivity. In conclusion, EDOF IOLs are efficient at providing distance and intermediate visions and safe with rare serious postoperative complications. Nevertheless, more clinical trails with randomized and controlled study designs and adequate duration are needed to clarify the tradeoffs between EDOF IOLs and other presbyopia-correcting IOLs.

## Data Availability

The datasets used and/or analyzed during the current study are available from the corresponding author on reasonable request.

## Abbreviations

AMO: Abbott Medical Optics
CI: Confidence interval
D: Diopter
EDOF: Extended depth of focus
FDA: Food and Drug Administration
IOLs: Intraocular lenses
logMAR: Logarithm of the minimum angle of resolution
NOS: Newcastle-Ottawa Scale
NRCS: Nonrandomized controlled studies
RCT: Randomized controlled trials
RR: Risk ratio
SD: Standard deviation
UDVA: Uncorrected distance visual acuity
UIVA: Uncorrected intermediate visual acuity
UNVA: Uncorrected near visual acuity
VA: Visual acuity
WMD: Weighted mean differences
